# Relationship between Trusting Behaviors and Psychometrics Associated with Social Network and Depression among Young Generation: A Pilot Study

**DOI:** 10.1371/journal.pone.0120183

**Published:** 2015-04-02

**Authors:** Motoki Watabe, Takahiro A. Kato, Alan R. Teo, Hideki Horikawa, Masaru Tateno, Kohei Hayakawa, Norihiro Shimokawa, Shigenobu Kanba

**Affiliations:** 1 School of Business, Monash University, Malaysia, Jalan Lagoon Selatan, 46150 Bandar Sunway, Selangor Darul Ehsan 46150, Malaysia; 2 Organization for Japan-US studies, Waseda University, Building No 120. 513, Waseda Tsurumaki-cho, Shinjuku-ku, Tokyo 1620041, Japan; 3 Department of Neuropsychiatry, Graduate School of Medical Sciences, Kyushu University, 3-1-1 Maidashi Higashi-ku, Fukuoka 8128582, Japan; 4 Brain Research Unit, Innovation Center for Medical Redox Navigation, Kyushu University, 3-1-1 Maidashi Higashi-ku, Fukuoka 8128582, Japan; 5 VA Portland Health Care System, 3710 SW US Veterans Hospital Road (R&D 66), Portland, Oregon 97239, United States of America; 6 Department of Psychiatry, Oregon Health & Science University, 3181 SW Sam Jackson Park Rd, Portland, Oregon 97239, United States of America; 7 Department of Neuropsychiatry, Sapporo Medical University, South 1, West 16, Chuo-ku, Sapporo 0608543, Japan

## Abstract

Maladaptive social interaction and its related psychopathology have been highlighted in psychiatry especially among younger generations. In Japan, novel expressive forms of psychiatric phenomena such as “modern-type depression” and “hikikomori” (a syndrome of severe social withdrawal lasting for at least six months) have been reported especially among young people. Economic games such as the trust game have been utilized to evaluate real-world interpersonal relationships as a novel candidate for psychiatric evaluations. To investigate the relationship between trusting behaviors and various psychometric scales, we conducted a trust game experiment with eighty-one Japanese university students as a pilot study. Participants made a risky financial decision about whether to trust each of 40 photographed partners. Participants then answered a set of questionnaires with seven scales including the Lubben Social Network Scale (LSNS)-6 and the Patient Health Questionnaire (PHQ)-9. Consistent with previous research, male participants trusted partners more than female participants. Regression analysis revealed that LSNS-family (perceived support from family) for male participants, and item 8 of PHQ-9 (subjective agitation and/or retardation) for female participants were associated with participants’ trusting behaviors. Consistent with claims by social scientists, our data suggest that, for males, support from family was negatively associated with cooperative behavior toward non-family members. Females with higher subjective agitation (and/or retardation) gave less money toward males and high attractive females, but not toward low attractive females in interpersonal relationships. We believe that our data indicate the possible impact of economic games in psychiatric research and clinical practice, and validation in clinical samples including modern-type depression and hikikomori should be investigated.

## Introduction

Culture and society have strongly influenced on human mental health and its disturbance [1]. The information technology revolution has rapidly changed a variety of social communication methods, which have strongly affected lifestyles and behaviors, especially among younger generations. Human communications have considerably shifted from direct to indirect contacts such as personal computers and mobile phones. In line with these changes, new types of psychiatric or behavioral disorders, such as Internet addiction, have emerged [2,3].

For example, novel expressive forms of psychiatric phenomena such as “modern-type depression (MTD)” and “hikikomori” have been reported especially among young people in Japan. MTD has been frequently reported in Japanese clinical settings [4,5], and seems to meet the criteria of major depressive disorder in ICD-10 and the DSM-IV, though no diagnostic tools have been established. Contrary to the classical prototype of depression in Japan, most MTD patients are young adults, showing less loyalty for social institutions, having negative feelings about social norms, a vague sense of omnipotence, and not being hard-working by nature [5,6]. “Hikikomori,” defined as a syndrome with six months or longer of severe social withdrawal, was also initially reported in Japan, and the prevalence rate has been reported as 1.2% in Japanese population [7]. The majority of patients are adolescents and young adults who become recluses in their parents’ homes for months or years [8]. They withdraw from contact with family, rarely have friends, and do not attend school or hold a job. An international vignette-used questionnaire survey indicates the spread of MTD and hikikomori in many other countries [9,10]. In general, people with these novel psychiatric syndromes have difficulty in communicating to others and establishing fruitful social interactions [10].

Psychiatric diagnostic tools including the Structured Clinical Interview for DSM-IV (SCID) have been established based on subjective interview, which are limited in grasping real-world social interactions. A previous survey suggests that both MTD and traditional type of depression are diagnosed as major depressive disorders by DSM-IV criteria, while the treatment strategies are quite different [10], which indicate the importance of differentiation between both phenotypes of depression. Novel systematic tools should be investigated to measure such aspects in psychiatry [11]. Economic games have been developed in the field of social psychology and economics, and have been proposed as a novel tool for evaluating interpersonal psychiatric problems. Clinical studies using economic games (prisoner's dilemma, the public goods game, the ultimatum game and the trust game) have revealed some difficulties of social decision-making in individuals with major depressive disorders and personality disorders [12–15].

The trust game, an economic game, has been widely used to evaluate a person’s trust toward others [16]. In this two-person game, the first player has to make a risky financial decision depending on how much s/he would trust the second player (partner). Recent studies have examined whether other factors such as personality and psychiatric conditions influence trusting behaviors and cooperation [14,15,17–19].

Trust is an essential ingredient of cooperative behaviors and an important factor in mental health. Trust allows people to establish cooperative relationships with others, resulting in socially supportive human relationships. Clinical case reports have indicated that people with MTD and hikikomori have difficulties in developing trust among family members, and colleagues in schools and working places [20–22]. We thus hypothesize that a common feature of modern psychiatric syndromes may be induced through difficulty in trusting others, and these features may not be limited to patients but also to the wider contemporary populations, especially the young.

As a first step in advancing this field of research, we herein conducted a trust game experiment with young Japanese samples to analyze how their trusting behaviors are affected by underlying psychosocial and personality aspects using various psychometric scales.

## Methods and Materials

This study has been approved by the IRB committee in Kyushu University (Approval Number 25–84).

### Participants

Participants were recruited by advertisement on a university campus. The experiment was conducted on July in 2013. Eighty-one Japanese students applied and participated. All the participants provided their written informed consent, which had been approved by the IRB committee, in advance to start the experiment. Structured diagnostic interviews for psychiatric disorders were not conducted for each participant, thus we could not exclude a possibility that some participants may have had psychiatric disorders.

### Procedure of Trust Game

**Fig 1:**
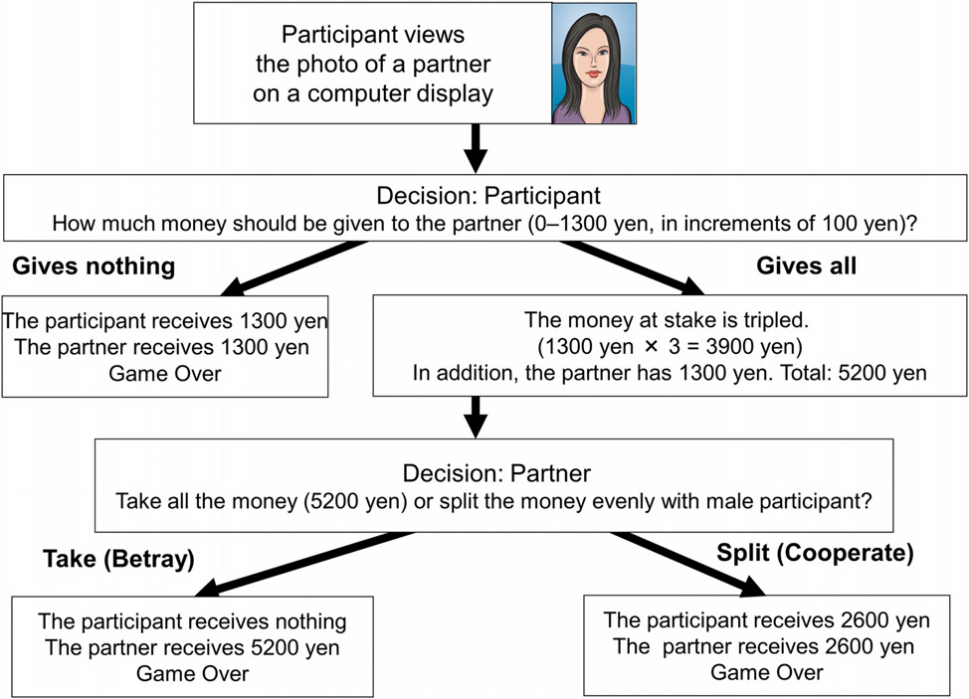
Trust Game Structure with the Most Extreme Cases.

Participants were individually instructed on the trust game, and answered a few example questions to confirm that they fully understood the game structure. Participants then made decisions regarding how much of 1,300 JPY (about 13 USD) to give to each of 40 photographed partners. The amount of money given (Monetary Scores) to each partner was tripled, and the partner then decided whether to split the money equally with the participant or to take the entire amount of the money. Fig. 1 shows the game flow of the two extreme cases. The participant’s decision regarding how much money to give to the partner was expected to function as a behavioral measure of the trust that the participant has toward the photographed partner. The only information about the partner was a neutral head-and-shoulders photograph of a similarly-aged male or female. After each decision, the participants evaluated how trustworthy and attractive the partner was.

### Photographed Partners

Prior to the present game experiment, 42 young males (mean age = 21.36, SD = .62) and 44 females (mean age = 20.88, SD = .66) of a similar age as the trust game participants were recruited using on-campus advertisements with the similar method as Watabe et al. [23]. Photographs including the head and shoulders with a neutral facial expression were taken with permission. We randomly selected 20 pictures (10 males and 10 females) to use in the present experiment. We also added 20 pictures of fashion models (10 males and 10 females) taken from a DVD with the same composition under permission by the publisher. This diversity of physical attractiveness was expected to have an effect on decisions in the trust game. All photographs are of Japanese.

After the experiment, each participant was asked if they knew each partner shown in the photographs. Five participants, who had known any of the photographed persons, were not included in the data analysis, in order to avoid confounding effects.

### Questionnaires

After playing the trust game, participants filled out self-rated scales including General Trust Scale (GTS), Internet Addiction Test (IAT), Lubben Social Network Scale (LSNS)-6, Patient Health Questionnaire (PHQ)-9, State-Trait Anxiety Inventory (STAI), Temperament and Character Inventory (TCI)-140, and Revised UCLA Loneliness Scale (UCLA-R). Previous studies reported that GTS, TCI, and STAI are related to decisions in trust games [18,24]. The GTS measures respondents’ estimation of others’ trustworthiness, a major factor in trust game with anonymous partners[24]. The reliability and validity of GTS have been confirmed across many countries [25]. The TCI-140 is a 140-item measure of seven basic personality dimensions [26], which has been validated in Japan [27]. STAI is a 40-item scale with two subscales namely State and Trait anxiety (20-item each) [28]. We used the Japanese version [29].

An international study has recently shown that LSNS-6 and UCLA-R scores of subjects with hikikomori are significantly different from healthy samples [30]. The LSNS-6 is a 6-item scale that assesses the number of people in an individual’s social network with whom one has social contact and social support [31]; which was validated in Japanese samples [32]. Originally, the LSNS was developed for elderly people, but it has been utilized in clinical research among youth in India [33–35]. Recent international hikikomori research has also shown that scores of LSNS-6 for individuals with hikikomori were much lower than normal controls, and individuals with hikikomori showed slightly higher scores on the family subscale than the friend subscale [30]. The above previous studies support the utilities of the LSNS-6 for younger generations. The UCLA-R is a 20-item measure that assesses how often individuals endorse subjective feelings of loneliness [36]; which was validated in Japanese samples [37].

Hikikomori, MTD and Internet use are suggested to be correlated [6]. Especially, an international questionnaire survey has suggested a positive link between Internet addiction and hikikomori [10]. Thus, we included PHQ-9 and IAT. PHQ-9, a 9-item measure of depressive symptoms, was originally developed by Kroenke et al. [38] and translated into Japanese [39]. PHQ-9 is widely used in clinical settings and community settings to assess depression-related symptoms [40]. The IAT is a 20-item measure of addictive use of the Internet [41]; which was validated in Japanese samples [42].

### Analysis

We analyzed the data of 81 university students (Male: N = 43, mean age = 20.44, SD = 1.67; Female: N = 38, mean age = 20.24, SD = 1.78) by SPSS Ver.22. All of them completed the game experiment, and were included in the analyses below.

We first perform ANOVA with correction for multiple comparisons (Bonferroni) for conforming consistency with the past studies [49, 50]. These studies report that male participants show more trusting behavior to others than female participants. Although we do not have hypothesis on gender difference of trusting behavior in this study, the conformation of the consistency would be worth to report. For ANOVA, based on gender and physical attractiveness score, we categorized the 40 photographs (20 females and 20 males) into four types (high-attractive-male, high-attractive-female, low-attractive-male, and low-attractive-female). Independent variables are gender (male, female) and attractiveness (high, low). Dependent variables are scores of the amount of money given (namely, “monetary score”), and the subjective level of trust to the partner (namely, “trusting rates”).

Next, we performed regression analysis with stepwise method by photograph type to analyze how the psychometric scales influence on trust behavior. We included 16 independent variables: trust to photographed partners, two LSNS subscale scores, seven subscales of TCI, total score of UCLA-R, total score of PHQ-9, two subscales of STAI, IAT total score, and sex of participants as a dummy variable. The dependent variable was the monetary scores for each type of photograph. To avoid the issue of multicollinearity, we chose stepwise option and confirmed that the scores of condition indexes were below 15 for each final model.

Third, we performed another regression analysis with more variables to analyze details by participants’ gender. While previous research has demonstrated that trust is a significant factor for cooperating with others, recent studies have suggested that gender differences exist for such cooperative decision-making [29,51,52]. We thus performed regression analysis with stepwise method by respondents’ gender. In this regression, 24 independent variables were included; IAT score, two subscale LSNS of scores, each item score of PHQ-9, two subscales of STAI scores, TCI-140 seven subscale scores, UCLA-R total score, trust to photographed partner (trusting rate), and living alone or not as a dummy variable. Again, to avoid multicollinearity, we chose stepwise option and confirmed that the scores of condition indexes were below 15 for each final model.

## Results

### ANOVA on Means Scores of Given Money and Trust to Partners

Table 1 summarizes mean scores of dependent variables for each type of photograph (high attractive males, low attractive males, high attractive females and low attractive females).

**Table 1:** Mean Ratio and SD of "Monetary Scores"(how much money given) and "Trusting Rates".

		Photographed Male Partner		Photographed Female Partner	
		High Attractiveness	Low Attractiveness	High Attractiveness	Low Attractiveness
**Male & Female Combined Participants N = 81**	**Ratio of Monetary Scores (SD)**	.292 (.196)	.361(.188)	.325 (.187)	.302 (.199)
	**Trusting Rates (from 0 to 9) (SD)**	3.38 (1.38)	3.62(1.48)	3.52 (1.24)	3.43 (1.37)
**Male Participants N = 43**	**Ratio of Monetary Scores (SD)**	.340 (.224)	.361 (.205)	.358 (.202)	.337 (.222)
	**Trusting Rates (from 0 to 9) (SD)**	3.63 (1.42)	3.86 (1.63)	3.54 (1.33)	3.60 (1.51)
**Female Participants N = 38**	**Ratio of Monetary Scores (SD)**	.237 (.142)	.265 (.153)	.287 (.164)	.262 (.163)
	**Trusting Rates (from 0 to 9) (SD)**	3.09 (1.30)	3.37 (1.26)	3.51 (1.13)	3.26 (1.17)

**Fig 2:**
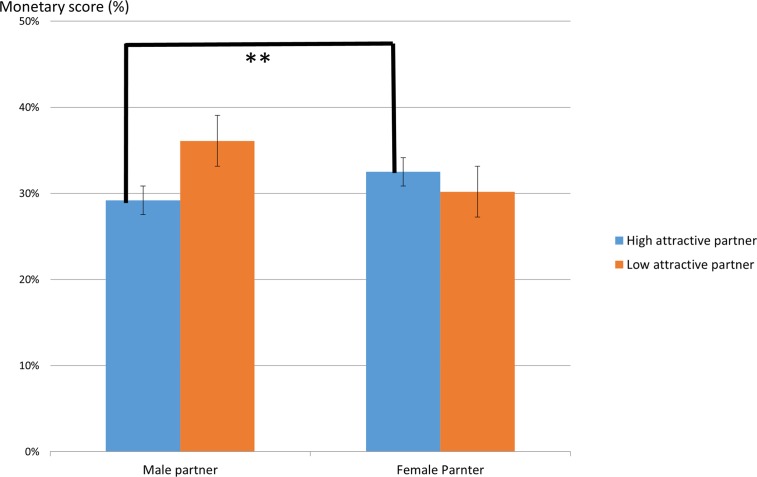
The ratio of monetary score given by partner’s sex and attractiveness (** p<.01).

For the monetary scores, consistent with previous research [43], male participants gave more money to partners than female participants (male participants: mean = .349, SE = .027, female participants: mean = .263, SE = .028) (F (1, 79) = 4.88, p<.05). A significant interaction was evaluated between photographed partner’s attractiveness and photographed partner’s sex (F (1, 79) = 6.77, p<.05) as shown in Fig. 2. Multiple comparison analysis revealed that a simple main effect of monetary scores between high attractive male and high attractive female was significant (p<.01).

**Fig 3:**
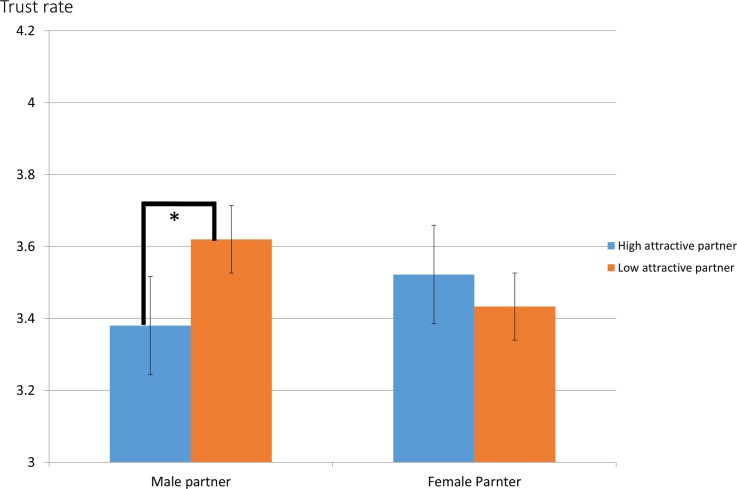
The ratio of trusting rates toward partner’s sex and attractiveness (*p<.05).

For the trust rates, although past research reports that male participants generally trusted partners more than female participants, the sex difference of trust level the this study was not significant (male participants = 3.67 (SE = .185); female participants = 3.32 (SE = .194)) (F (1, 79) = 1.62, p = .21). A significant interaction was evaluated between photographed partner’s attractiveness and photographed partner’s sex (F (1, 79) = 5.24, p<.05). Multiple comparison analysis revealed that a simple main effect of trust rates between high and low attractive male partners was significant as shown in Fig. 3 (p<.05). The above results are consistent with previous research [43,44].

### Regression Analysis with male-female combined data

We included the 16 independent variables mentioned above. The results are summarized in Table 2. We revealed that “how much the participants trust the photographed person” was the significant independent variable for each type of photograph. We have analyzed every possible set of independent variables.

**Table 2:** Results of Regression Analysis (Monetary Scores as Dependent Variable).

	Photographed Male Partner		Photographed Female Partner	
Independent Variable	High Attractiveness	Low Attractiveness	High Attractiveness	Low Attractiveness
	Beta	Beta	Beta	Beta
**Trusting Rates**	.617**	.700**	.587**	.642**
**Model**	*N = 70*, *AdjR2 = .372*, *F(1*, *69) = 42*.*41*, *p<*.*001 Condition Index = 5*.*345*	*N = 70*, *AdjR2 = .482*, *F(1*, *69) = 66*.*14 p<*.*001 Condition Index = 5*.*349*	*N = 70*, *AdjR2 = .285*, *F(1*, *69) = 36*.*20*, *p<*.*001 Condition Index = 6*.*202*	*N = 70*, *AdjR2 = .404*, *F(1*, *69* *) = *48.47*, *p<*.*001 Condition Index = 5*.*373*

Note:

* p<.05,

** p<.01.

Eleven participants were excluded in this analysis for failing to complete the questions.

### Regression Analysis by participant gender

Next, we performed another regression analysis with the 24 independent variables mentioned above. The results are summarized in Table 3. For all photograph types, trusting rates showed consistently high predictability of monetary scores. The more the participants trusted the partners, the higher amount of money the participants gave.

**Table 3:** Results of Regression Analysis with Stepwise option (Monetary Scores as Dependent Variable).

		Photographed Male Partner		Photographed Female Partner	
	Independent Variable	High Attractiveness	Low Attractiveness	High Attractiveness	Low Attractiveness
		Beta	Beta	Beta	Beta
**Male Participants**	**Trusting Rates**	.536**	.720**	.541**	.588**
	**LSNS Family**	-.326*	-.230*	-.316*	-.324**
	**Living Alone (Dummy Yes = 1, No = 0)**	-	-	.274*	.246*
	**Model**	*N = 35*, *AdjR* ^*2*^ = .*452*, *F(2*, *33) = 15*.*44*, *p<*.*001 Condition Index = 9*.*999*	*N = 35*, *AdjR* ^*2*^ = .*581*, *F(2*, *33) = 25*.*30*, *p<*.*001 Condition Index = 8*.*701*	*N = 35*, *AdjR* ^*2*^ = .*467*, *F(3*, *32) = 11*.*20*, *p<*.*001 Condition Index = 9*.*681*	*N = 35*, *AdjR* ^*2*^ = .*554*, *F(3*, *32) = 15*.*50*, *p<*.*001 Condition Index = 9*.*275*
**Female Participants**	**Trusting Rates**	.675**	.619**	.625**	.594**
	**Item 8 of PHQ-9**	-.318*	-.314*	-.308*	-
	**Model**	*N = 33*, *AdjR* ^*2*^ = .*451*, *F(2*, *31) = 14*.*55*, *p<*.*001 Condition Index = 5*.*486*	*N = 33*, *AdjR* ^*2*^ = .*400*, *F(2*, *31) = 12*.*01*, *p<*.*001 Condition Index = 6*.*425*	*N = 33*, *AdjR* ^*2*^ = .*410*, *F(2*, *31) = 12*.*46*, *p<*.*001 Condition Index = 7*.*034*	*N = 33*, *AdjR* ^*2*^ = .*332*, *F(1*, *32) = 17*.*42*, *p<*.*001 Condtion Index = 5*.*819*

Note:

* p<.05,

** p<.01.

Eight male participants and five female participants with incomplete data were excluded in this analysis.

Although most scales and subscales were not selected as significant independent variables for trusting behaviors, we found that LSNS-family for male participants and item 8 of PHQ-9 (psychomotor agitation and/or retardation) for female participants were selected as a significant independent variable. (The scatter plots of these variables is shown in S1 Fig.) Interestingly, male participants with higher scores on LSNS-family showed lower monetary scores across all the types of photographs. The higher they have support from family, the less trust they show to others. Among male participants, living alone was also a significant predictor only for female photographs. Male participants living alone gave more to female partners than male participants living with somebody.

The results for female participants were same as those for male participants in that trust to photographed partner was consistently significant for trusting behaviors across all the types of photographs. However, there were differences between male and female participants. The LSNS-family and living alone were not significant for the female participants. Instead, item 8 of PHQ-9 (agitation and/or retardation) was a significant independent variable for trusting behaviors. Female participants with higher scores on item 8 showed lower monetary scores toward males and high attractive females, but not toward low attractive females.

## Discussion

In the present pilot study, we have specified the psychometrics associated with trusting behaviors among young generation. One of hikikomori-related measures (LSNS-family) and item 8 of PHQ-9 (subjective agitation and/or retardation) proved to be significant predictors for trusting behaviors in university students.

### Scores of Given Money and Trust to Partners

In the present study, male participants showed more trusting behaviors than female participants. This result is consistent with previous research [49]. We also found that attractive males tend to be less trusted. Recent evolutionary psychology literature has suggested that attractive males tend to be dominant over unattractive males in competition for mate selection and procreation [45]. Thus, females may be more vigilant of attractive males, which may explain the reasons why such males are not trusted.

### Social Network and Trusting Behaviors

In the present study, a significant association between LSNS-family and trusting behaviors was found in male participants. LSNS shows the degree of perceived supports from family, friends, and neighbors. LSNS-family refers to the support from family. The present results suggest that more family support leads to less trust to others in males. This is consistent with arguments by political scientists [46], and social psychologists [43,47]. Fukuyama claimed that businesses based on strong ties among family members hardly extend their business beyond kin relationships. Kiyonari and Yamagishi have argued that commitment relationships inside of a group tend to reduce trust level toward the people outside the group [48]. De Dreu et al. has demonstrated that oxytocin facilitates cooperation and trust toward ingroup members, but it also reduces them toward outgroup members [47]. In addition, a recent epidemiological study focusing on hikikomori has shown that hikikomori is more likely to occur with the family of educated parents than with the family of relatively uneducated parents [49]. This epidemiological data [49] may also be consistent with the present study. To explain the epidemiological finding, Umeda et al. reported that highly educated parents have higher incomes and can afford to have their children jobless at home [49] by citing Furlong’s sociological analysis [50]. Based on these previous reports, a possible interpretation of the present data is that strong support from family (ingroup) may reduce trust level for pictured persons (outgroup). This tendency may also provide a possible reason for the occurrence of hikikomori syndrome. When family members worry about a person with hikikomori and try to take care of the person, his heavy dependence on family (which is called AMAE in Japanese [51]) results in the reduction of the level of trust outside of family members. Finally, the person may become more dependent on his family members, and his social withdrawnness may be reinforced. This vicious circle would create hikikomori syndrome. Hikikomori is known to be much more prevalent for males than females [7]. Moreover, a recent international cross-sectional study has shown that scores of LSNS for individuals with hikikomori were much lower than normal controls, and individuals with hikikomori showed slightly higher scores on the family subscale than the friend subscale [30]. These results partially support our hypothesis based on the present analysis. Further studies should be conducted to clarify this aspect.

On the other hand, the present study has shown that, compared with male participants living with somebody, male participants living alone gave higher amounts of money to female partners, not to male partners. This result was significant even when the above LSNS-family’s effect was statistically controlled. One possible explanation of this finding is that males living alone may tend to seek female partners. According to evolutionary psychology, “gift giving” is one means to attract opposite-sex partners and especially males are likely to perform “gift giving” more than females [45]. Previous experimental research has also shown that males’ giving behaviors were affected by partner’s physical attractiveness when the partner was female [23]. Thus, this “living alone” effect could be explained outside of trusting behaviors. Rather, it would be a “gift giving” behavior to attract opposite-sex partners.

### Item 8 of PHQ-9

The validity of PHQ-9 has been discussed in various different populations [52–55]. Forkmann et al. (2013) reported that “the item 8-removed version” shows better predictability of depression than the original PHQ-9 with elder patient samples [52]. Has item 8 lost value in modern psychiatry? Interestingly, the present data may give a novel significant value to item 8. Item 8 measures psychomotor agitation and/or retardation by asking “*Moving or speaking so slowly that other people could have noticed*. *Or the opposite being so fidgety or restless that you have been moving around a lot more than usual*.” Importantly, agitation/retardation, rated by PHQ-9, shows a “subjective” agitation/retardation but not “observed” agitation/retardation. To diagnose major depressive disorders, retardation and agitation should be assessed by not the first person but observers. Thus, we should consider the differences between “subjective agitation/retardation (by PHQ-9)” and “observed agitation/retardation”. Our data may propose a novel role of “subjective” agitation in psychiatry. Based on the PHQ-9, we could not distinguish which of these tendencies (agitation or retardation, or both) is actually associated with trusting behaviors. In the previous clinical studies of depression, males have been found to have more retardation than females, while females have been found to have more agitation than males [56]. Thus, we suppose that, in the present females’ samples, agitation might be much more prevalent than retardation, and subjective agitation may be critical in females’ trusting behaviors. The present results indicate that females with high subjective agitation may tend to make more defensive and cautious decisions in daily life, which may cause difficulties in various social interactions. We prospect that subjective agitation in young females may link to recent psychiatric conditions such as social withdrawal and modern depression in society today [6]. Subjective retardation may also link to trusting behaviors and the above-mentioned modern psychiatric pathologies. Further investigations are needed to examine how agitation and/or retardation affect trusting behaviors, using separated scales of each aspect in clinical settings.

### Depression and Economic Games

Economic games such as prisoner's dilemma, the public goods game, the ultimatum game and the trust game have been conducted among individuals with major depressive disorders [12–14]. Clark et al. reported a significant association between depression and several economic games including trust game [14]. They reported that symptoms of depression are correlated only with behaviors in prisoners’ dilemma and public goods game, not with those in the trust game and ultimatum game. On the other hand, the present data has suggested that item 8 of PHQ-9 (subjective agitation and/or retardation) is associated with trust game behavior while we found no association with rest of items and total score of PHQ-9. Our results are partially consistent with the study by Clark et al. [14]. Considering these two previous studies used different depression scales (DASS and PHQ-9) with different samples in different cultures, further investigation should be conducted.

### Limitation

In the present pilot study, we did not utilize psychiatric structural interviews such as SCID. Thus, we did not exclude individuals with psychiatric disorders, and some participants might have had psychiatric disorders. Without diagnostic interviews, we did not conclude to diagnose whether some participants have psychiatric disorders or not. Future trials should include diagnostic interviews to solve this limitation. In addition, to validate the findings from the present pilot study, additional experiments with larger samples and different populations in different countries are needed. Self-rated questionnaires limit the reliability of the answers. Item 8 of PHQ-9 asks both subjective agitation and retardation at the same time, which limits the differentiation between them. Clinical data including individuals with MTD and hikikomori should be collected to confirm the utilities of trust game in psychiatry.

## Conclusion

In sum, the present pilot study has demonstrated that, for males, support from family was negatively associated with cooperative behavior toward non-family members. In addition, females with higher subjective agitation and/or retardation gave less money toward males and high attractive females, but not toward low attractive females in interpersonal relationships. We believe that the present pilot study has indicated the possible impact of economic games in psychiatric research and clinical practice in the future, and further studies should be investigated in clinical samples including “modern-type depression” and hikikomori.

## Supporting Information

S1 Fig Scatter plots of Monetary Scores with significant variables. (TIF)
