# Supplementary figures and images for: Relationship between Trusting Behaviors and Psychometrics Associated with Social Network and Depression among Young Generation: A Pilot Study
Source: PLoS One. 2015 Apr 2;10(4):e0120183. doi: 10.1371/journal.pone.0120183 (PMC4383339; doi:10.1371/journal.pone.0120183)

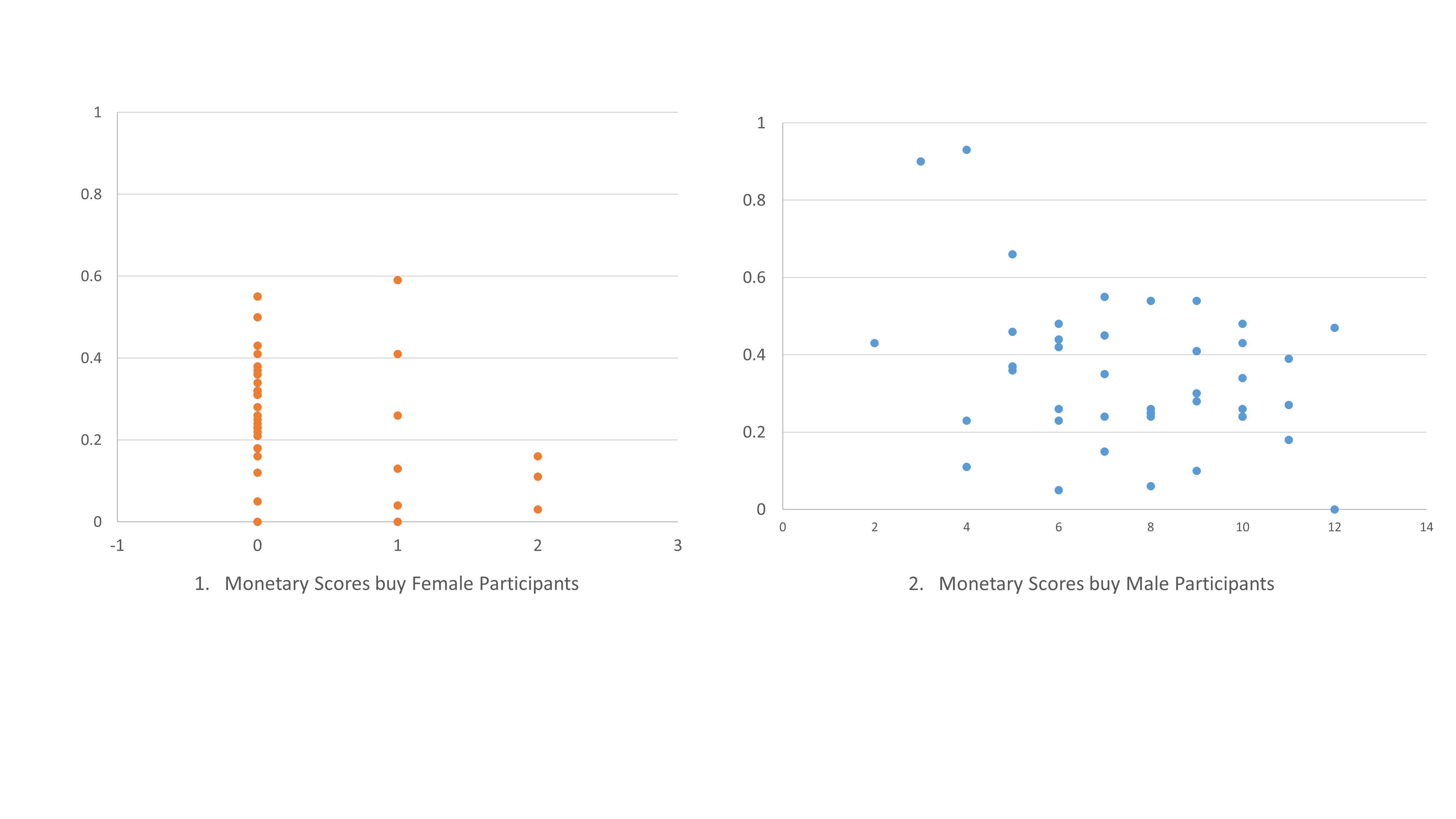

Supplement: S1 Fig — (TIF) [file pone.0120183.s001.tif]
